# Design Parameters Affecting the Performance of Vortex-Induced Vibration Harvesters

**DOI:** 10.3390/mi16020122

**Published:** 2025-01-22

**Authors:** Alberto Pasetto, Michele Tonan, Federico Moro, Alberto Doria

**Affiliations:** Department of Industrial Engineering, University of Padova, 35131 Padova, Italy; alberto.pasetto.2@phd.unipd.it (A.P.); michele.tonan@unipd.it (M.T.); federico.moro@unipd.it (F.M.)

**Keywords:** vortex, vibrations, energy harvesting, piezoelectric, harvester, damping

## Abstract

Vortex-induced vibration harvesters are usually equipped with small piezoelectric patches mounted near the cantilever clamp, where the largest longitudinal stress occurs. This paper, aiming to improve energy harvesting performance, investigates the possibilities of extending the patch length and modifying the length and mass of a bluff body mounted on a harvester to induce vortex shedding. A novel analytical model based on dimensionless numbers is presented to determine the output voltage generated by a cantilever harvester subjected to periodic vortex shedding. This model highlights the design parameters having the largest influence on harvester performance and provides guidance to the planning of experimental tests and the interpretation of experimental results. Some prototype harvesters with different designs are built. First, experimental tests are carried out to identify the natural frequencies and damping ratios of the prototypes; then, the prototypes are tested in a wind tunnel to assess energy harvesting performance. The best performance is achieved when the patch length is about 20% of the cantilever length, the bluff body is long, and its mass reaches the minimum value. This result agrees with the prediction of the model.

## 1. Introduction

Vortex-induced vibrations (VIVs) have been studied for many years due to their negative effects on the safety of machines and structures [1]. The influence of the geometric and mechanical properties of the vibrating system was studied to minimize the effect of excitation due to vortex shedding [2,3,4]. The most dangerous phenomena occurred in lightly damped structures [5] where the increase in damping was considered a positive factor to reduce the amplitude of vibrations [6,7].

Recent developments in vibration energy harvesting technologies have opened up the possibility of exploiting fluid-excited vibrations to harvest energy [8]. This possibility appears to be very useful for remote sensors that are powered by small photovoltaic panels. In adverse weather conditions, solar energy production decreases [9] and can be complemented by wind [10] and rain energy harvesting [11,12] to guarantee the minimum energy level needed for sensors and data storage. A wind flow impinging on an elastically mounted body can produce vibrations due to different phenomena [1], which can be exploited for energy harvesting. Experimental and numerical studies on vibrations generated by vortex shedding from cylinders were reported in [13,14]. Galloping harvesters exploiting the interaction between wind and non-cylindrical bodies were studied in [15,16]. An inverted flag harvester exploiting a flutter phenomenon was presented in [17], and finally, harvesters exploiting turbulence were proposed in [18].

The present research focuses on vortex-induced vibration because, after proper natural frequency tuning, it is effective in the range of small flow velocities, which are very common in the field of civil and industrial structures. In contrast, harvesters based on dynamic instabilities (galloping and flutter harvesters) require the wind velocity to be higher than the cutoff velocity [19]. The turbulence harvester also produces larger amounts of energy when the wind velocity increases.

Vortex-induced vibration harvesters typically consist of one or more piezoelectric patches attached to a structural layer, which is equipped with a cylindrical bluff body to induce vortex shedding [13]. Various layouts were proposed in the literature, such as the I-shaped configuration, in which the axes of the bluff body (a cylinder) and the cantilever are aligned, and the T-shaped configuration, in which the axes are perpendicular [20]. More complex layouts are described in [21,22]. VIV harvesters achieve the best performance when the vortex shedding frequency is equal to the natural frequency of the harvester. This resonance condition is made complex by the nonlinear dynamics of near-wake vortex shedding. When the frequency of vortex shedding is close to the natural frequency of the system, a considerable interaction between flow and structure is present, and a range of flow velocities with large-amplitude oscillations named the “lock-in region” appears [23]. Many studies focused on the fluid dynamics of vortex shedding and the lock-in phenomenon. A detailed experimental analysis of the effect of the cylinder aspect ratio on both vortex shedding and harvested energy was presented in [4]. The effect of the ratio between cylinder length and cantilever width was studied experimentally in [24]. Computational fluid dynamics (CFD) simulations of a VIV harvester were presented in [25]. The effect of electrical load on the output power of the harvester was studied by many researchers, who considered simple resistors connected to piezoelectric patches [14,25].

Despite such great research effort, some aspects of the mechanical design of a T-shaped VIV harvester are still worth studying. Most of the above-mentioned studies have considered cantilevers equipped with small piezoelectric patches placed in the best position, that is, near the cantilever clamp where the largest longitudinal stress occurs. The effect of extending the length of the piezoelectric patch has been scarcely addressed in the literature. The extension of the patch may have unexpected results: on the one hand, it can increase the charge generated and the energy stored; on the other hand, a larger patch area glued to the structural layer increases damping, limiting the dynamics of the harvester and its performance.

The cylindrical bluff body can be regarded as a tip mass added to the end of the cantilever harvester [26]. The increase in the tip mass has a predictable effect consisting in the lowering of the natural frequency of the system and, in turn, of the lock-in velocity. However, some researchers [14,21] reported other effects of tip mass, such as significant variations in the maximum vibration amplitude and the size of the velocity window in the lock-in region. Other researchers highlighted the effect of the tip mass on the shape of higher-order modes [11]. These phenomena are explored in this paper.

This paper is organized as follows: In Section 2, a mathematical model is presented showing the effect of the main design parameters on harvester performance under ideal conditions. The final equations are expressed in dimensionless terms by making use of the Skop–Griffin number, which is widely used in the field of VIV. In Section 3, experimental tests for the identification of the dynamic parameters necessary to compare the performances of different harvesters are presented. The damping increase due to the bonding of long piezoelectric patches is highlighted. In Section 4, wind harvesting tests carried out on harvesters equipped with patches having different lengths and with different cylindrical bluff bodies are presented. The experimental results are discussed in light of the model in Section 2, and the limits of simplified models are discussed. Finally, conclusions are drawn in Section 5.

## 2. Mathematical Model of the VIV Harvester

**Figure 1:**
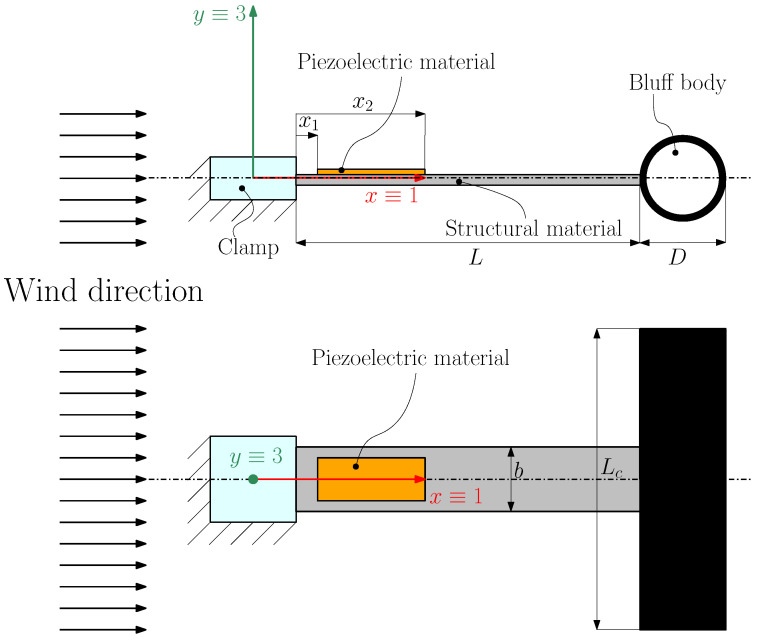
Scheme of the piezoelectric harvester equipped with a cylindrical bluff body.

Figure 1 shows a scheme of the T-shaped piezoelectric harvester investigated in this paper to exploit VIV. The three main components of the harvester are the structural layer, the piezoelectric layer, and the cylindrical bluff body. The bluff body has two different functions: the first is to lower the first natural frequency of the cantilever because it is a mass concentrated on the tip of the cantilever, and the second is to generate vortex shedding phenomena. According to [1], when the cylinder is subjected to a constant airflow, vortexes are shed with a frequency ($f_{s}$) given by the following relationship:

(1) $$f_{s}=\mathrm{St}\;\frac{U}{D}$$

where *U* is the wind velocity, *D* is the external diameter of the cylinder, and St is the Strouhal number.

On the one hand, by properly selecting the diameter of the cylinder (*D*), it is possible to adjust the vortex shedding frequency ($f_{s}$) to match the natural frequency of the harvester at a specific wind velocity (*U*). On the other hand, when the cylinder diameter is fixed, it is possible to adjust the natural frequency of the harvester modifying cantilever stiffness and tip mass to match the vortex shedding frequency at a certain wind velocity.

The cantilever beam consists of an aluminum beam that is 130 mm long, with a rectangular section (21 mm width, 1 mm thickness). The piezoelectric patch is a Macro Fiber Composite (MFC) built by Smart Material GmbH (Dresden, Germany) and exploits the $d_{31}$ effect. The longitudinal direction 1 corresponds to the *x* axis, and the transverse direction 3 corresponds to the *y* axis in Figure 1. It is a wafer structure with rectangular piezoceramic rods sandwiched between layers of adhesive, electrodes, and polyimide [27].

**Figure 2:**
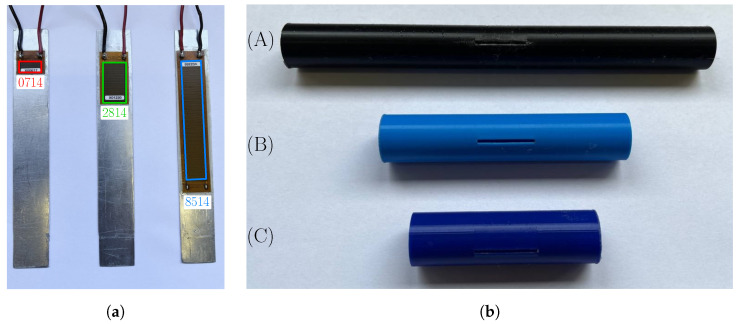
Test samples: (**a**) patches of different lengths bonded to aluminum cantilevers; (**b**) cylindrical bluff bodies of different lengths (A—178 mm, B—94 mm, and C—65 mm).

In this work, three different patches manufactured by Smart Material Corporation were glued to the structural layer (Figure 2). The patches, which have the same width (14 mm) and different lengths (7 mm, 28 mm, and 85 mm), were labeled with the codes “0714”, “2814”, and “8514”, respectively (see Figure 2a). The cylindrical bluff body is made of 3D-printed plastic material. The bluff body has an external diameter D= 19 mm, while the length and thickness of the cylinder were varied in the experimental tests.

In the harvester layout considered in this work, the bluff body does not rotate about a pivot like in [28], but it moves owing to the elastic deformation of the cantilever. Generally speaking, cantilever deformation gives the cylindrical bluff body a circular path [4]. Nevertheless, the experimental results presented in [4] showed that in typical T-shaped harvesters, the displacement along the flow (*x* direction in Figure 1) is much smaller than the cross-flow displacement (*y* direction). Therefore, the cylindrical bluff body can be considered a system vibrating in the cross-flow direction as in [14,19].

### 2.1. Electromechanical Model

The origin of the Cartesian coordinate system x,y in Figure 1 is located at the clamped end (base). The cylindrical bluff body, placed at the free end of the beam (x=L), is modeled as a tip mass $M_{t}$. Metal electrodes are located on the top and bottom sides of the piezoelectric patch to collect the electric charge generated by the piezoelectric effect due to strain; electrodes are connected to the power conditioning circuit through terminals. For simplicity, a 1D model of the VIV harvester is considered, neglecting the displacement in directions *x* and *z*. It is also assumed that the piezoelectric patch does not cover the entire surface of the beam, but rather a delimited area represented by the interval $\left[x_{1},x_{2}\right]$.

According to [29], the electrical output of the piezoelectric patch can be analytically modeled assuming that the patch is very thin compared with the structural layer; therefore, the mechanical response of the cantilevered beam is basically unchanged. The analytical model used here for the assessment of the energy harvesters is described below.

The absolute transverse displacement *w* of any point of the beam can be obtained by solving the following equation of motion [30]:

(2) $$\frac{\partial^{2}M\left(x,t\right)}{\partial x^{2}}+c_{s}I\;\frac{\partial^{5}w\left(x,t\right)}{\partial x^{4}\partial t}+c_{a}\frac{\partial w(x,t)}{\partial t}+m\frac{\partial^{2}w\left(x,t\right)}{\partial t^{2}}=F\left(t\right)\delta \left(x-L\right)$$

where *t* is time, *M* is the bending moment, $c_{s}$ is the equivalent coefficient of strain rate damping, *I* is the equivalent area moment of inertia of the composite cross section, $c_{a}$ is the viscous air damping coefficient, *m* is the mass per unit length of the beam, *F* is a generic lumped force applied to the cantilever tip, and δ is the Dirac delta. The presence of the clamp and the tip mass is taken into account by setting proper boundary conditions at the ends of the beam according to Equation C.12 and Equation C.13 in [31].

If the piezoelectric patch does not cover the whole beam, the bending moment reads as follows:

(3) $$M\left(x,t\right)=EI\;\frac{\partial^{2}w\left(x,t\right)}{\partial x^{2}}+\theta \;v\left(t\right)\;\left[H\left(x-x_{1}\right)-H\left(x-x_{2}\right)\right]$$

where EI is the bending stiffness of the composite cross section, *v* is voltage, and *H* is the Heaviside function. In Equation (3), the electromechanical coupling coefficient is defined as

(4) $$\theta =\frac{e_{31}\;b}{2\;h_{p}}\;\left(h_{b}^{2}-h_{c}^{2}\right)$$

where $e_{31}$ is the piezoelectric constant, *b* is the width of the beam, $h_{p}$ is the thickness of the piezoelectric layer, $h_{b}$ is the position of the bottom of the piezoelectric layer from the neutral axis, and $h_{c}$ is the position of the top of the piezoelectric layer from the neutral axis.

According to the modal expansion approach, the absolute displacement can be expanded into an absolutely and uniformly convergent series as follows:

(5) $$w\left(x,t\right)=\sum_{n=1}^{\infty }\Psi_{n}\left(x\right)\;\eta_{n}\left(t\right)$$

where the eigenfunction $\Psi_{n}$ is the *n*th mode of vibration (function of the spatial coordinate *x*) and $\eta_{n}$ is the *n*th modal displacement (function of time *t*). The modes of vibration fulfill the boundary conditions (clamped end, tip mass at the free end). These are obtained by solving the eigenvalue problem related to Equation (2) and are normalized by setting the tip amplitude to one $\Psi_{n}\left(L\right)=1$. In this way, $\eta_{n}\left(t\right)$ corresponds to the tip displacement due to the *n*th mode.

Inserting Equation (5) in Equation (2), assuming proportional damping, and exploiting the orthogonality of eigenfunctions, the dynamic equation for the *n*th mode becomes

(6) $$m_{n}\;{\ddot{\eta }}_{n}+c_{n}\;{\dot{\eta }}_{n}+\left(k_{n}+\frac{\varphi_{n}^{2}}{C_{p}}\right)\eta_{n}=f_{n}$$

where $m_{n}$ is the modal mass, $c_{n}$ is the modal damping coefficient, and $k_{n}$ is the modal stiffness of the cantilever, $\varphi_{n}$ is the forward modal coupling coefficient, and $f_{n}$ is the modal force for the *n*th mode [31]. The modal parameters can be calculated as follows:

(7) $$m_{n}=\int_{0}^{L}\Psi_{n}\left(x\right)\;m\Psi_{n}\left(x\right)\;dx+\Psi_{n}\left(L\right)M_{t}\Psi_{n}\left(L\right)+\frac{d\Psi_{n}\left(x\right)}{dx}{|}_{x=L}I_{t}\frac{d\Psi_{n}\left(x\right)}{dx}{|}_{x=L}$$

(8) $$k_{n}=\int_{0}^{L}\frac{d^{2}\Psi_{n}\left(x\right)}{dx^{2}}EI\frac{d^{2}\Psi_{n}\left(x\right)}{dx^{2}}dx$$

(9) $$f_{n}=F\left(t\right)\Psi_{n}\left(L\right)$$

According to the proportional damping assumption, modal damping is defined as

(10) $$c_{n}=2\;\zeta_{n}\sqrt{k_{n}m_{n}}$$

where $\zeta_{n}$ is the modal damping ratio, which can be found by experimental testing.

For the unimorph harvester layout shown in Figure 1, the forward modal coupling coefficient is equal to the backward modal coupling coefficient [31], yielding

(11) $$\varphi_{n}=\theta \;\left(\frac{d\Psi_{n}\left(x\right)}{dx}{|}_{x=x_{2}}-\frac{d\Psi_{n}\left(x\right)}{dx}{|}_{x=x_{1}}\right)$$

The vibrations induced by the vortex are transformed into electric charge as a result of the piezoelectric effect and stored in the collector electrodes as in a capacitor. In this case, the electric charge at the positive electrode of the harvester, with surface Γ, is the flux of the electric displacement D, that is,

(12) $$q=\int_{\Gamma }D\cdot n\;d\Gamma$$

where n is the unit normal vector of Γ, directed opposite to the *y* axis. Assuming a thin piezoelectric patch, the electric displacement is constant on Γ and has only a transverse component, that is,

(13) $$D_{3}=e_{31}\;S_{1}+\varepsilon_{33}^{S}\;E_{3}$$

where $S_{1}$ is the component of the axial strain, $E_{3}$ is the transverse component of the electric field, $e_{31}$ is the piezoelectric stress charge coefficient, and $\varepsilon_{33}^{S}$ is the permittivity at a constant strain. Note that the longitudinal component of the strain can be approximated for a thin patch as [30]

(14) $$S_{1}=-h_{pc}\;\frac{\partial^{2}w\left(x,t\right)}{\partial x^{2}}$$

where $h_{pc}$ is the distance of the center of the piezoelectric patch cross section from the neutral axis, and

(15) $$E_{3}=-\frac{v(t)}{h_{p}}$$

where v(t) is the electric voltage. By inserting Equations (14) and (15) in Equation (13) and by noting that $D\cdot n=-D_{3}$, the electric charge (12) becomes

(16) $$q\left(t\right)=b\int_{x_{1}}^{x_{2}}\left(e_{31}\;h_{pc}\;\frac{\partial^{2}w\left(x,t\right)}{\partial x^{2}}+\varepsilon_{33}^{S}\;\frac{v(t)}{h_{p}}\right)\;dx$$

Due to the charge conservation law applied to the positive terminal, the output current (with sign convention coming out from the positive terminal) results in

(17) $$i\left(t\right)=-\frac{dq(t)}{dt}$$

After integrating Equation (16) over time, the output current becomes:

(18) $$i\left(t\right)=-e_{31}\;b\;h_{pc}\left(\frac{\partial w(x,t)}{\partial x\;\partial t}{|}_{x=x_{2}}-\frac{\partial w(x,t)}{\partial x\;\partial t}{|}_{x=x_{1}}\right)-C_{p}\frac{dv(t)}{dt}$$

where the piezoelectric patch capacitance is defined as

(19) $$C_{p}=b\;\varepsilon_{33}^{S}\;\frac{x_{2}-x_{1}}{h_{p}}$$

Using Equations (5) and (11), the output current can be rewritten as

(20) $$i\left(t\right)=\sum_{n=1}^{\infty }\varphi_{n}\;\frac{d\eta_{n}\left(t\right)}{dt}-C_{p}\frac{dv(t)}{dt}$$

Assuming an open-circuit operation (no current flows through the terminals, that is, i(t)=0), the open-circuit voltage (OCV) can be obtained from the time integration of Equation (20) as

(21) $$v_{0}\left(t\right)=v_{0}\left(t_{0}\right)+\frac{1}{C_{p}}\;\sum_{n=1}^{\infty }\varphi_{n}\;\eta_{n}\left(t\right)$$

where $v_{0}\left(t_{0}\right)$ is the OCV at the initial time instant $t_{0}$. The OCV is used here to characterize the electrical performance of the vibration harvester. In fact, in practical applications, power electronic converters for energy extraction are used most of the time under open-circuit conditions to minimize energy loss, and the electric charge is extracted from the harvester when the maximum voltage level (or the maximum stored energy) is reached [32].

The electric potential energy stored in the harvester (that is, the electric work spent to charge electrodes at voltage $v_{0}\left(t\right)$) can be calculated as in a capacitor [33] as follows:

(22) $$E_{e}\left(t\right)=\frac{1}{2}\;C_{p}\;v_{0}^{2}\left(t\right)$$

The average energy stored can thus be obtained as

(23) $$E_{e_{avg}}=\frac{1}{2}\;C_{p}\;v_{0_{RMS}}^{2}$$

where $v_{0_{RMS}}$ is the root mean square (RMS) value of the OCV.

### 2.2. Aerodynamic Force

When the cylinder is subjected to a constant airflow, vortexes are shed with a frequency given by Equation (1). The periodic shedding of vortexes results in a variation of pressure surrounding the cylinder and, hence, in a time-dependent force orthogonal to the flow direction (lift). As the first approximation, the effect of cylinder motion on vortex shedding can be neglected and the time-dependent lift is modeled as a harmonic lift applied to the cylinder axis [34] as follows:

(24) $$F_{a}\left(t\right)=\frac{1}{2}\;\rho DL_{c}U^{2}C_{L}\left(t\right)$$

where ρ is the fluid density, $L_{c}$ is the length of the cylinder, $C_{L}\left(t\right)=C_{L0}e^{i\omega_{s}t}$ is the time-dependent lift coefficient, and $\omega_{s}=2\pi f_{s}$.

The electromechanical model in Section 2.1 holds for a generic force F(t) applied to the cantilever tip and cannot be directly used if $F_{a}\left(t\right)$ is applied at the center of the bluff body (that is, at D/2 distance from the tip). However, the model can be generalized to adding a torque $\tau \left(t\right)=D/2\;F_{a}\left(t\right)$ applied to the tip. It can be shown that, in this case, the modal forcing term becomes [14]

(25) $$f_{n}=\chi_{n}\;F_{a}\left(t\right)$$

where

(26) $$\chi_{n}=\Psi_{n}\left(L\right)+\frac{D}{2}\frac{d\Psi_{n}\left(x\right)}{dx}{|}_{x=L}$$

is the modal constant of the *n*th mode.

The harmonic force model does not include the effect of the cylinder motion on the aerodynamic force. Other models introduce additional equations to take into account the coupling between the lift coefficient and the cylinder motion. For example, the model presented by Facchinetti et al. in [23] uses the van der Pol oscillator equation, with a forcing term that depends on the acceleration of the cylinder, to calculate $C_{L}\left(t\right)$. In this work, Equation (24) is adopted to model the vortex shedding lift because it allows a direct interpretation of many experimental results. Equation (24) cannot model the nonlinear interaction between cylinder and fluid motion and the extension of the lock-in region.

### 2.3. Dimensionless Harmonic Solution

The VIV harvesters considered in the present work were used with wind velocities of less than 5 m/s. Considering the typical values of the Strouhal number for cylinders, the vortex shedding frequency belongs to the range 10–25 Hz and can be tuned to the first natural frequency of the cantilever harvester. The excitation of higher-order modes is negligible, and the dynamic response of the VIV harvester can be studied only by considering the contribution of the first mode of Equation (6). If only the first mode is considered and the OCV at $t_{0}$ is zero, Equation (21) becomes

(27) $$v_{0}\left(t\right)=\frac{1}{C_{p}}\;\varphi_{1}\;\eta_{1}\left(t\right)$$

The dynamic equation of the first mode considering the modal forcing term defined by Equation (25) becomes

(28) $$m_{1}{\ddot{\eta }}_{1}+c_{1}{\dot{\eta }}_{1}+\left(k_{1}+\frac{\varphi_{1}^{2}}{C_{p}}\right)\eta_{1}=\chi_{1}\;F_{a}\left(t\right)$$

A time-harmonic solution is sought for Equation (28) by setting $F_{a}\left(t\right)=F_{a0}\;e^{i\omega_{s}t}$, $\eta_{1}\left(t\right)=\eta_{10}\;e^{i\omega_{s}t}$, where $\eta_{10}$ is the complex amplitude of the response of the first mode. This results in

(29) $$\eta_{10}=\frac{\chi_{1}\;F_{a0}}{m_{1}\left(\omega_{1}^{2}+2i\zeta_{1}\omega_{1}\omega_{s}-\omega_{s}^{2}\right)}$$

where $\omega_{1}=\sqrt{\left(k_{1}+\varphi_{1}^{2}/C_{p}\right)/m_{1}}$ is the natural frequency of the first mode taking into account the electromechanical coupling and $\zeta_{1}=c_{1}/\left(2m_{1}\omega_{1}\right)$ is the modal damping ratio of the first mode. From Equation (29), the amplitude of the modal response at resonance ($\omega_{s}=\omega_{1}$) can be derived as follows:

(30) $$|\eta_{10}|=\frac{\chi_{1}\;F_{a0}}{m_{1}2\zeta_{1}\omega_{1}^{2}}$$

By expressing Equation (27) in the frequency domain and by substituting Equation (24) in Equation (30), the OCV amplitude can be calculated as

(31) $$\begin{array}{c} |v_{0}|=\frac{1}{C_{p}}\varphi_{1}\frac{\chi_{1}}{m_{1}2\zeta_{1}\omega_{1}^{2}}\frac{1}{2}\rho L_{c}DC_{L0}U^{2} \end{array}$$

Equation (31) can be expressed in dimensionless form, since many experimental studies on VIV have shown a correlation between the amplitude of cylinder vibrations and the Skop–Griffin number, which is defined as [4,35,36]

(32) $$\mathrm{SG}=2\pi^{3}{\mathrm{St}}^{2}m^{*}\zeta_{1}$$

where $m^{*}$ is the mass ratio defined as the ratio between the vibrating mass and the mass of fluid having the same volume of the cylinder. In the present case, considering the modal damping of the first mode $\zeta_{1}$ and the modal mass of the first mode, the mass ratio is

(33) $$m^{*}=\frac{4\;m_{1}}{\pi \rho D^{2}L_{c}}$$

and the Skop–Griffin number becomes

(34) $$\mathrm{SG}=\frac{8\pi^{2}{\mathrm{St}}^{2}\zeta_{1}m_{1}}{\rho L_{c}D^{2}}$$

The amplitude of the OCV generated by the harvester at resonance can be expressed as a function of the Skop–Griffin number by substituting $U=\frac{\omega_{1}D}{2\pi St}$ in Equation (31) and performing some algebraic manipulations as follows:

(35) $$|v_{0}|=\frac{1}{2C_{p}}\varphi_{1}DC_{L0}\frac{\chi_{1}}{\mathrm{SG}}$$

On the right-hand side of Equation (35), the term $\frac{\varphi_{1}D}{C_{p}}$ has the physical meaning of voltage generated by a displacement equal to cylinder diameter; hence, the OCV can be normalized with respect to this value obtaining the following dimensionless equation:

(36) $$|v_{0}|\frac{C_{p}}{\varphi_{1}D}=\frac{C_{L0}}{2}\frac{\chi_{1}}{\mathrm{SG}}$$

Equation (36) is useful because it defines the main parameters that determine the voltage at the harvester terminals in the presence of a harmonic lift. In particular, the dimensionless voltage is proportional to the lift coefficient and the modal constant and inversely proportional to the Skop–Griffin number (SG). In turn, SG depends on the mass ratio $m^{*}$, modal damping $\zeta_{1}$, and Strouhal number St.

### 2.4. Calculated Results

The mathematical model presented in the previous section shows that the extension of the piezoelectric patch influences the generated voltage through the forward coupling coefficient $\varphi_{1}$, capacitance $C_{p}$, and modal damping $\zeta_{1}$. The last parameter appears in the Skop–Griffin number. The mass of the piezoelectric patch, which is a small fraction of the mass of the harvester (including the structural layer and the cylinder), has a small effect on $m_{1}$, which appears in SG. The small stiffness of the piezoelectric patch has a negligible effect on the modal constant $\chi_{1}$.

To highlight only the effect of patch length on the performance of a harvester excited by a generic tip force (regardless of its nature), an open-loop analysis was carried out. In this way, the feedback effect on the aerodynamic force by the motion of the cylinder was neglected.

The best location for a piezoelectric patch covering only a small portion of the structural layer is near the clamp, where the second spatial derivative of the first mode (corresponding to the cantilever curvature) reaches the highest value. Therefore, the coordinate $x_{1}$ (see Figure 1), which defines the starting point of the piezoelectric patch, was kept small and constant ($x_{1}=$ 8 mm), while the coordinate $x_{2}$, which defines the end point of the patch, was varied. Additionally, modal damping was parametrically varied since an increased extension of the piezoelectric patch and the glued area increases damping.

**Figure 3:**
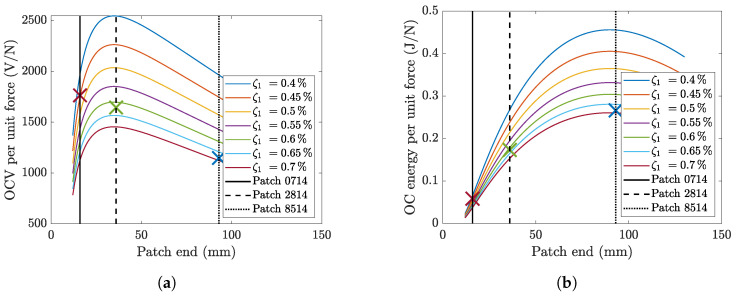
(**a**) Effect of patch length on the OCV and (**b**) the energy generated by the cantilever harvester according to the electromechanical model per unit tip force applied at resonance. Patch start: $x_{1}=$ 8 mm. The crosses (red—0714, green—2814, blue—8514) represent the calculated OCV and energy using the measured damping of the configurations in Section 3.

Figure 3a shows the open-circuit voltage. A unitary harmonic force (1 N) was applied to the cylinder axis, and the RMS value of the OCV was calculated. The vertical lines in Figure 3a represent the lengths of the three tested patches. Figure 3b shows the average energy calculated according to Equation (23) considering a unitary harmonic force applied to the cylinder axis.

The calculated results highlight that an increase in the damping ratio leads to a large decrease in the performance of the VIV harvester in terms of both OCV and energy. When the piezoelectric patch is short, the increase in length increases the OCV, which reaches a maximum. A further increase in length leads to a monotonic decrease in the RMS value of the OCV. The extension of the piezoelectric patch length has a positive effect on energy for a wide range of lengths (see Figure 3b). For very large lengths (comparable with cantilever length), a further increase in length leads to a decrease in energy.

In conclusion, the main performance indexes of the harvester (OCV and energy) show a maximum value at a certain length. This optimal length depends not only on the spatial derivative of the modal shape (Equation (11)) and the capacitance (Equation (19)), but also on the increase in damping caused by the piezoelectric patch and the adhesive. The latter effect can only be analyzed experimentally.

## 3. Experimental Characterization of the Harvester by Means of Impulsive Tests

Experiments were performed to measure the damping ratio of the first bending mode of the harvesters.

**Figure 4:**
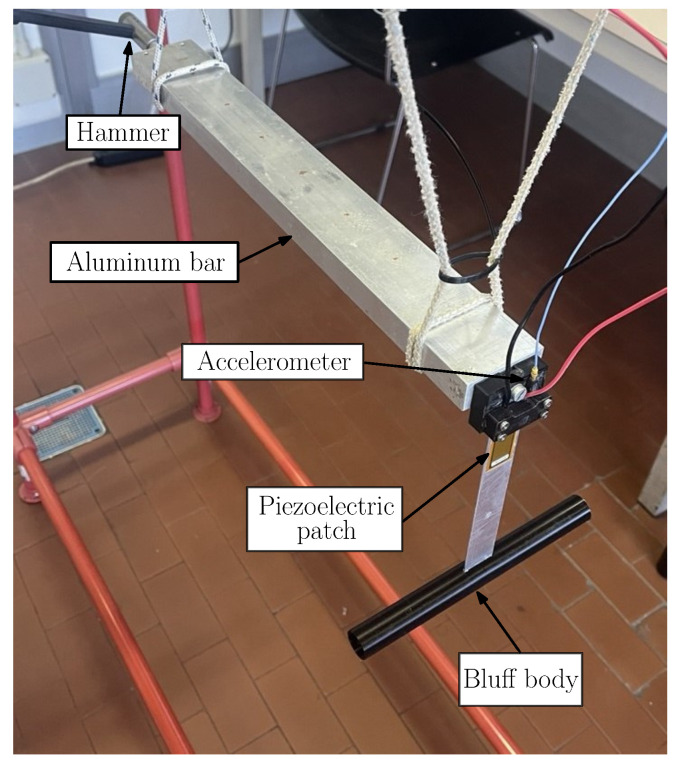
Experimental setup for the impulsive characterization of the harvesters.

Each harvester was fixed to one extremity of a suspended aluminum bar; the suspension was adopted to isolate the system from environmental vibrations [37]. An impulsive force was applied to the other extremity of the aluminum bar with a hammer for modal testing (PCB 086D03, sensitivity 2.25 mV/N) to excite the harvester. The acceleration of the base of the harvester was measured using a PCB 352C22 accelerometer (sensitivity 10.48 mV/g). The cantilever was fixed with its tip down; in this way, it was in a configuration of stable equilibrium. The experimental setup is shown in Figure 4.

The voltage generated by the piezoelectric patch, the accelerometer signal, and the hammer signal were acquired at a sampling frequency of 2048 Hz with an NI9230 module. The sensors are manufactured by PCB Piezotronics (Depew, NY, USA) whereas the module is manufactured by NI National Instrument Corporation (Austin, TX, USA). Since the internal resistance of the acquisition module is 324 kΩ, the piezoelectric patches directly connected to the module would work under load. Therefore, an additional resistor (10 MΩ) was connected in series with the module to increase the resistance of the circuit and simulate the open-circuit condition.

Experimental and numerical results showed that the first modal displacement dominates the response of the system [14,26]; hence, tip displacement is given by

(37) $$w\left(L,t\right)\approx \Psi_{1}\left(L\right)\;\eta_{1}\left(t\right)$$

Since Equation (27) shows that also the OCV is proportional to the modal displacement of the first mode ($\eta_{1}$), it results that the OCV is proportional to the tip displacement.

Data were analyzed using MATLAB R2024a software. The logarithmic decrement method was applied to identify the damping ratio from the time record of measured voltage. In the frequency domain, the frequency response function (FRF) between the OCV and base acceleration was calculated.

First, three harvesters with the same geometry and different patches were tested. To investigate the linearity of the harvesters, each harvester was tested by applying base accelerations with peak values of 2, 3, 6, and 9 g. Five acquisitions of 6 s were performed for each harvester and for each excitation level.

**Figure 5:**
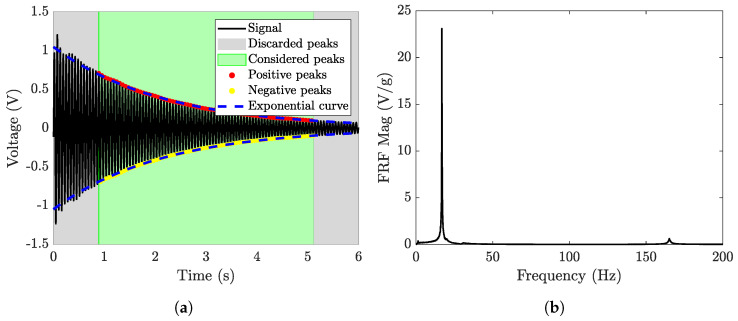
(**a**) Example of voltage signal (hammer hit 3 g, patch 2814, cylinder with mass 10 grams, and length 178 mm) with peaks used for the calculation of the damping ratio (**a**), and (**b**) example of FRF (magnitude) between the OCV and the base acceleration.

For damping calculation, the voltage signal of each test was filtered to remove small fluctuations due to higher-order modes. The first part of the time record, which includes some irregularities, and the last part of the time record (with amplitudes smaller than 10% of the initial amplitude) were discarded. Then positive and negative peaks were found (see Figure 5a), and the logarithmic decrement method was applied between the peaks at a distance of 10 periods. The calculation was repeated for all the positive peaks and for all the negative peaks, finding the damping ratio for each cycle of 10 periods. Finally, the mean damping ratio between cycles was calculated for each test.

In addition, the non-filtered voltage signal was used to compute the FRF; the result is shown in Figure 5b. The FRF shows a large resonance peak at the first natural frequency of the cantilever (16.8 Hz), while the peaks at other natural frequencies (e.g., at 165.2 Hz) are negligible, demonstrating that only the first mode of vibration influences the generated voltage.

**Figure 6:**
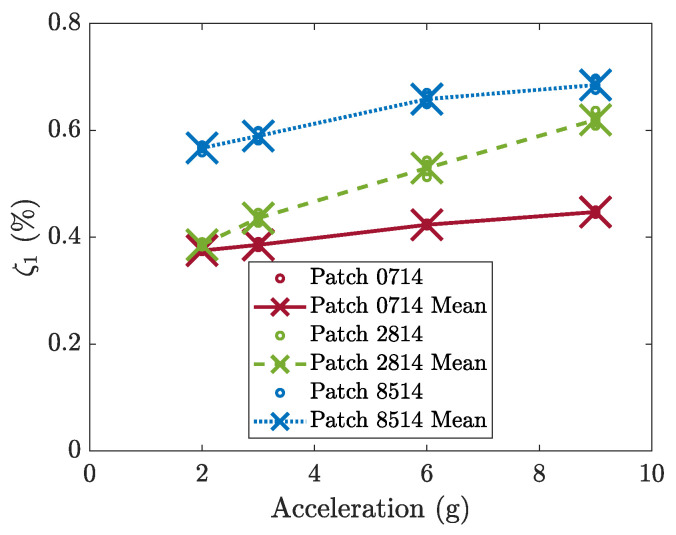
Damping of the three patches obtained with different excitation amplitudes (2 g, 3 g, 4 g, and 9 g).

The damping ratios of the three patches measured for increasing excitation levels are reported in Figure 6. The three patches show an increase in damping for larger excitations; this nonlinearity is almost negligible for patches 0714 and 8514 and more important for patch 2814. The natural frequencies and damping ratios of the three harvesters excited by 9 g acceleration impulses are summarized in Table 1.

Second, the effect of the cylinder length on the damping ratio was investigated, since variations in cylinder length may alter aerodynamic damping. To perform this analysis, cylinders with different lengths but with the same mass (10 grams (hereafter gr)) and the same external diameter (19 mm) were mounted on the cantilever equipped with patch 2814, which was tested with the impulsive method, as shown in Figure 4.

**Figure 7:**
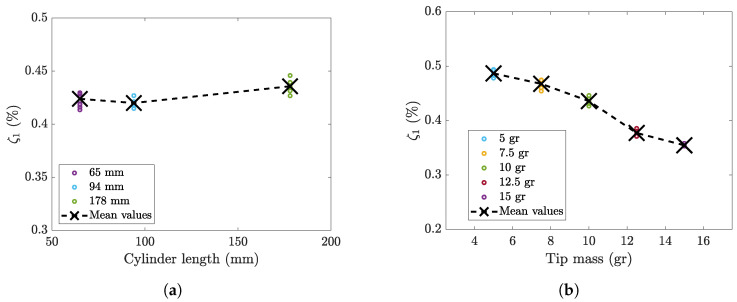
(**a**) Damping values of the same cantilever (with patch 2814) equipped with three cylinders with different lengths, and (**b**) the same cantilever equipped with five cylinders with different masses, both with an excitation amplitude of 3 g.

Five tests were carried out for each cylinder. The amplitude of the excitation was set to be equal to 3 g. The damping ratios were calculated as in the previous tests, and the results are shown in Figure 7a. The damping ratio is quite constant if the length of the cylinder is equal to 65 and 94 mm, whereas when the length increases, there is a slight increase in the damping ratio. In fact, when the surface area of the cylinder increases, there is a larger interaction between the bluff body and the fluid, which causes a larger dissipation. However, this increase is rather small and can be considered negligible.

Finally, five harvesters equipped with patch 2814 and cylinders of different masses were tested with the same procedure. The external diameter (19 mm) and the length of the cylinders (178 mm) did not change, and only the internal diameter was changed. In this way, the effect of the cylinder mass on the damping ratio was measured. The excitation amplitude was chosen to be equal to 3 g in these tests as well. The measured damping ratios are reported in Figure 7b. As expected, the increase in the mass of the cylinder caused a decrease in the damping ratio, and this behavior can be easily understood by considering the damping ratio formulated as $\zeta_{1}=c_{1}/\left(2\sqrt{k_{1}m_{1}}\right)$. In this equation, the stiffness $k_{1}$ and the damping coefficient $c_{1}$ are constant because the structure is the same, while the mass $m_{1}$ varies because the tip mass changes.

## 4. Effects of the Design Parameters of the Harvester in Wind Tunnel Experiments

### 4.1. Experimental Equipment—Wind Tunnel

**Figure 8:**
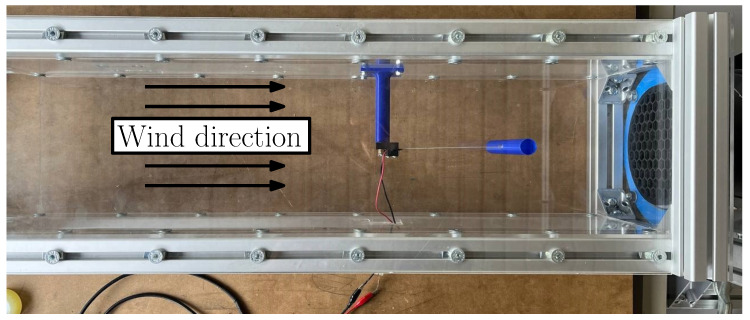
VIV harvester inside the wind tunnel (top view).

To evaluate the performance of the harvesters, a wind tunnel was adopted to create a steady airflow (see Figure 8). This wind tunnel comprises a channel with a rectangular cross section, measuring 19 cm in width and 20 cm in height, and extends 80 cm in length. Airflow is generated by a fan located at the channel end, allowing for wind velocities up to 4 m/s. Within the tunnel, the harvesters are securely mounted on a slender vertical column placed upwind from the cylinder. The axis of the cylindrical bluff body is parallel to the direction of gravity, so this setup minimizes gravitational effects on the harvester’s performance during testing.

The maximum blockage ratio of the wind tunnel experiments is about 9% (considering the cylinder with a length of 178 mm); thus, the blockage effect has negligible effects on the VIV phenomenon [7]. Another important parameter is the gap between the ends of the cylinder and the walls of the wind tunnel. It results in 58% of the cylinder diameter. This value is large enough to avoid the wall effect, even taking into account the presence of the boundary layer on the walls of the wind tunnel [24,38].

The OCV was acquired by means of a 9230 NI board with a sampling rate of 2048 Hz and a total number of samples of 20,480 (10 s). Five acquisitions were performed at each wind velocity.

**Figure 9:**
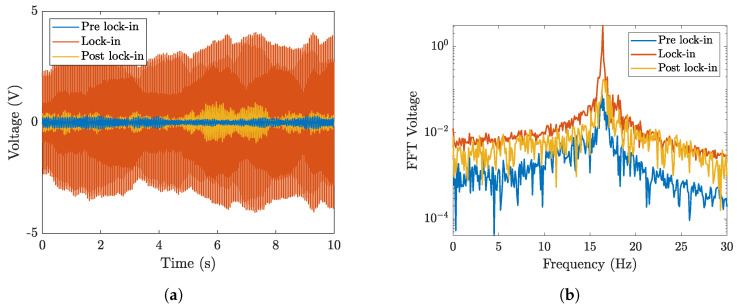
(**a**) Time domain OCV pre lock-in (1.4 m/s), at lock-in (2.1 m/s), and post lock-in (2.7 m/s) for patch 2814 with a 10 gr cylinder (length 178 mm), and (**b**) the corresponding FFTs of the three voltage signals.

Figure 9a shows typical time records of the OCV measured at different wind velocities. These curves were obtained using a cantilever equipped with a piezoelectric patch 2814 and a cylindrical bluff body (length 178 mm, diameter 19 mm, mass 10 gr).

The three curves depicted in Figure 9a represent the OCV at velocities lower than the lock-in region (pre lock-in), at lock-in, and at velocities higher than the lock-in region (post lock-in). The lock-in phenomenon occurs when the vortex shedding frequency reaches the natural frequency of the cantilever harvester. When the wind velocity is smaller than the lock-in value, the amplitude of vibrations is small and the OCV is low. When the harvester works in the lock-in region, the OCV reaches large values close to 4 V due to the large amplitude of vibrations. When the harvester operates above the lock-in region, the voltage strongly decreases and the waveform becomes irregular with many beats.

Figure 9b shows the fast Fourier transform (FFT) of the OCV in the three conditions. It confirms the excitation of the first mode and its strong predominance in the lock-in region.

### 4.2. Effect of Patch Length

The harvesters equipped with the different patches characterized in Section 3 were tested in the wind tunnel. All patches were bonded to the cantilever with the active part of the patch starting at a distance of $x_{1}=8$ mm from the clamp.

**Figure 10:**
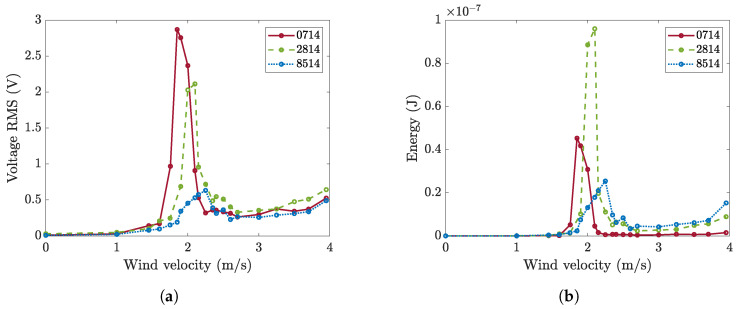
Wind tunnel tests: (**a**) RMS value of OCV generated by the three prototypes with different patches, and (**b**) corresponding energy.

The root mean square (RMS) of the OCV generated by each patch in the wind tunnel is shown in Figure 10a (the mean RMS between five acquisitions is shown). Figure 10b shows the average collected energy calculated according to Equation (23). Both the OCV and energy show the lock-in phenomenon. When the wind velocity is lower than the value that makes the vortex shedding frequency equal to the natural frequency, the response is small and increases slightly with *U*. Then there is a large increase in amplitudes, and these amplitudes remain large for a velocity band: this is the lock-in phenomenon. For higher velocities, the vibration amplitude suddenly decreases. In this range, amplitudes are larger than in the pre-lock-in, and this effect is due to the increased turbulence at higher velocities.

The analysis of Figure 10a highlights that the lock-in velocity slightly increases as the length of the patch increases. This effect is due to the extension of the patch that slightly increases the stiffness of the cantilever. The RMS value of the OCV decreases as the length of the piezoelectric patch increases. This result agrees with the calculations reported in Figure 3a if the transition between the curves corresponding to the damping values identified for the different harvesters is considered and if the wind force is assumed to be constant (the crosses in Figure 3a correspond to the tested harvesters). Figure 10b shows that the average energy reaches a peak when the harvester is equipped with a medium-length piezoelectric patch (2814). This result is in reasonable agreement with the calculations reported in Figure 3b if the different damping ratios of the harvesters are considered.

**Figure 11:**
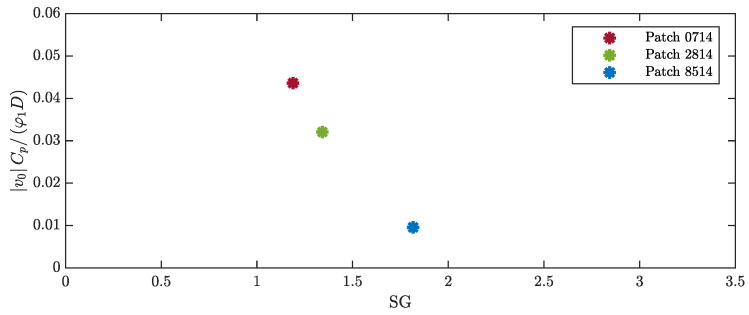
Dimensionless voltage against Skop–Griffin number for harvesters with different patches.

The measured OCV can be expressed in a dimensionless form as a function of the Skop–Griffin number and the other dimensionless parameters, as shown in Section 2.3. The modal constant $\chi_{1}$ is not affected by the length of the patch, which has a negligible effect on the modal shape. In addition, the lift coefficient $C_{L0}$ does not depend on the length of the patch. For the calculation of the Skop–Griffin number, the modal mass was calculated, the damping ratio was identified as described in Section 3, and the St number was calculated from the lock-in velocity and frequency. The results, which are presented in Figure 11, show that the extension of the patch length leads to an increase in the Skop–Griffin number, which is primarily caused by the increase in the damping ratio. The dimensionless voltage decreases as the SG number increases. It should be noted that many researchers on VIV harvesting reported that the dimensionless displacement of the cylinder decreases as SG increases [36], and the dimensionless voltage used in the present study is proportional to the dimensionless displacement.

### 4.3. Effect of Cylinder Length

Wind tunnel experiments on harvesters equipped with the same 2814 piezoelectric patch but with three cylinders of different lengths (see Figure 2b) were carried out. The external diameter and the mass were the same for the three cylinders; the cylinder thickness was varied to keep the cylinder mass constant. Therefore, the three prototypes had the same natural frequency, while the aspect ratio (AR) of the cylinder (which is the ratio between cylinder length and diameter), the ratio between the width of the cantilever and the length of the cylinder, and the mass ratio were different. These parameters are summarized in Table 2.

**Figure 12:**
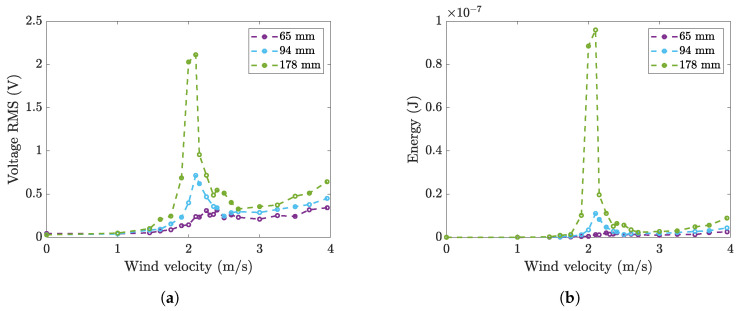
Wind tunnel tests: (**a**) RMS value of OCV generated by patch 2814 using three cylinders with different lengths, and (**b**) corresponding energy.

The results of this analysis are reported in Figure 12. Figure 12a shows the RMS value of the OCV, whereas Figure 12b shows the average collected energy. Experimental results show that the RMS value of the OCV monotonically decreases as the cylinder length decreases. The value of lock-in velocity is the same for the long cylinder (D= 178 mm) and the medium cylinder (D= 94 mm) and is slightly larger for the short cylinder (D= 65 mm). The force $F_{a}$ exerted by the fluid near wake on the cylinder can be considered a fluctuating lift force [23]. Therefore, if the fluid velocity *U* and the lift coefficient $C_{L}$ were constant, the force $F_{a}$ would depend linearly on the projected area *A* of the cylinder perpendicular to the direction of the fluid velocity. In the present case, since the diameter *D* was kept constant during tests, $F_{a}$ would linearly depend on $L_{c}$ as well. Consequently, Equation (31) shows that the voltage would be proportional to the cylinder length. Actually, Figure 12a shows that, when cylinder length decreases from 178 to 94 mm (the ratio between projected areas being 0.54), there is a larger decrease in the generated voltage, which varies from 2.1 to 0.7 V (the ratio being 0.33). Additionally, when cylinder length decreases further from 94 to 64 mm (the ratio between projected areas being 0.69), the voltage decrease is larger than the decrease in the projected area.

These measured trends can be explained by considering some phenomena that have been highlighted by previous research. In 2016, Akaydin et al. [24] showed that vortex shedding is strongly reduced when the aspect ratio tends to 1, because most of the airflow passes over the cylinder ends rather than over the cylindrical surface. In 2018, Azadeh et al. [4] experimentally investigated the effect of the flow around the free ends of the cylinder on the wake that generates the fluctuating force. They reported variations in the Strouhal number depending on the AR. In addition, they found that a decrease in AR from 10 to 5 caused an increase in the generated voltage and the extent of the lock-in region, whereas a further decrease in AR below 2.5 caused the disappearance of VIV. Unfortunately, they carried out the tests on cylinders having different AR and natural frequency; hence, the lock-in took place for rather different values of velocity. Therefore, it is difficult to distinguish the effect of AR from the effect of velocity in their results.

When the cantilever clamp was located downstream of the incoming airflow, some researchers found a splitter plate effect [4,24,39]. In fact, the cantilever surface prevented the formation of part of the wake and caused a large reduction in VIV.

The experimental results presented in this research refer to prototypes with the cantilever clamp mounted upstream of the incoming airflow and having AR in the range 3.4–9.4. On the one hand, the splitter plate effect is not present. On the other hand, it is possible that with this flow configuration, even if AR is rather large (AR=9.4), the reduction in AR causes a reduction in generated voltage. This effect adds to the effect of area reduction, and both lead to a large reduction in voltage when AR decreases.

**Figure 13:**
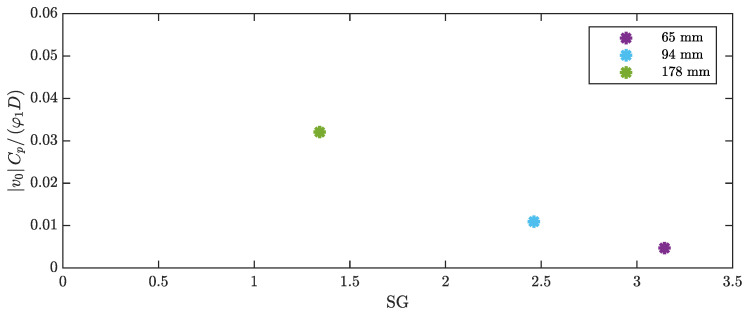
Dimensionless voltage against Skop–Griffin number for harvesters with different cylinder lengths.

Additionally, for the tests with different cylinder lengths, the measured OCV can be normalized and expressed as a function of dimensionless parameters (Equation (36)). It should be noted that, since the various cylinders have the same tip mass, the modal constant ($\chi_{1}$) does not depend on cylinder length, whereas the lift coefficient $C_{L0}$ may change due to the different cylinder AR. The Skop–Griffin number strongly increases as the length of the cylinder decreases due to the increase in mass ratio and is scarcely influenced by modal damping, which weakly depends on cylinder length. Figure 13 shows that the dimensionless voltage decreases as SG increases.

### 4.4. Effect of Cylinder Mass

The last series of tests aimed to study the effect of the tip mass (cylinder mass) on OCV and energy. To isolate the effect of the tip mass, the piezoelectric patch (2814), the cantilever beam, and the external geometry of the cylinder were kept constant in all prototypes. The constancy of the external geometry guaranteed an unchanged flow pattern about the non-vibrating cylinder. The variation in tip mass was achieved by modifying the internal diameter of the cylinder. Table 3 summarizes the mass ratios and natural frequencies of these prototypes.

**Figure 14:**
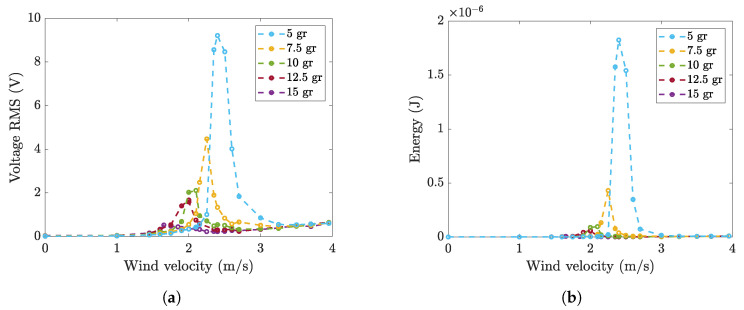
Wind tunnel tests: (**a**) RMS value of OCV generated by patch 2814 using five cylinders with different masses, and (**b**) the corresponding energy.

Figure 14a shows the RMS value of the OCV generated by the piezoelectric patch 2814 using five cylinders with different masses. The RMS value of the lock-in OCV increases strongly as the cylinder mass decreases. If the cylinder mass halves (passing from 10 gr to 5 gr), the RMS value becomes roughly four times larger. The decrease in cylinder mass also increases the extension of the lock-in region; this effect is consistent with previous research on VIV, which showed an increase in the extension of the lock-in region as the vibration amplitude increased [4]. Figure 14b shows the energy against the wind velocity. The effect of varying the mass of the cylinder on the energy is even more pronounced since the latter depends on the square of the RMS value of the OCV.

**Figure 15:**
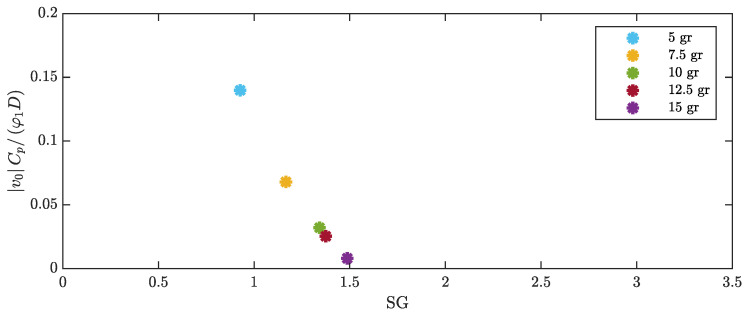
Dimensionless voltage against the Skop–Griffin number for harvesters with different cylinder masses.

The effect of cylinder mass can be studied by expressing the dimensionless voltage as a function of the dimensionless parameters defined in Section 2.3. When the mass of the cylinder changes, the SG number changes due to the large variations in the mass ratio, the variations in $\zeta_{1}$, and the variation in St (see Equation (34)). Since the modes of vibration ($\Psi_{n}\left(x\right)$) used in the mathematical model that leads to Equation (36) are normalized by setting the tip amplitude to 1 (which is the maximum amplitude of the first mode), the parameters $\chi_{1}$ and $\varphi_{1}$ are not affected by the value of the tip mass. The lift coefficient $C_{L0}$ should not change because the flow geometry is constant. Figure 15 shows that SG increases when tip mass increases and that dimensionless voltage decreases when SG increases.

## 5. Conclusions

Most of the VIV harvesters presented in the literature consider a small patch mounted near the clamped end of the structural layer. The idea of extending the length of the active piezoelectric layer to improve the open-circuit voltage and the harvested energy was investigated both analytically and experimentally in this work. This was motivated by the practical consideration that power electronic converters used for the energy management of vibration harvesters operate primarily under no-load conditions. A novel mathematical model has been proposed that shows the dependence of the open-circuit voltage at resonance on a few relevant dimensionless parameters, such as the Skop–Griffin number, the modal constant, and the lift coefficient. The calculated results have shown that an increase in the damping ratio leads to a large decrease (about 50%) in the performance of the VIV harvester, evaluated in terms of OCV. A series of impulsive tests were carried out to analyze the effect of the main parameters of the piezoelectric patch and the cylindrical bluff body on damping. In addition, a series of wind tunnel experiments were carried out to analyze the effect of the main parameters affecting the OCV. The use of dimensionless quantities in the voltage equation has proven to be a powerful tool for the analysis of experimental results due to the reduction in the number of model parameters. Experimental results have shown that the extension of patch length leads to an increase in the Skop–Griffin number, which is chiefly caused by the increase in damping. Experimental tests have shown that modal damping is weakly influenced by the aerodynamic effects related to cylinder length. Therefore, when the cylinder mass is constant, reducing the length of the cylinder leads to a strong increase in the Skop–Griffin number because the mass ratio increases. Finally, when the mass of the cylinder changes, the Skop–Griffin number chiefly changes due to the large variations in the mass ratio and, in particular, increases as the mass of the cylinder increases. The Skop–Griffin number increase leads in turn to a reduction in the dimensionless voltage. Therefore, energy harvesting can be improved by designing harvesters equipped with a medium-length patch and a long and light cylinder.

## Figures and Tables

**Table 1:** Summary of the natural frequencies and damping ratios of the three harvesters equipped with three different patches and the same cylindrical bluff body (length 178 mm, diameter 19 mm, mass 10 grams). These values of the damping ratios refer to the tests carried out with an excitation level of 9 g.

	Patch 0714	Patch 2814	Patch 8514
$f_{n}$ (Hz)	16.0	16.8	19.2
$\zeta_{1}\;\left(\%\right)$	0.45	0.62	0.68

**Table 2:** Geometrical parameters and mass ratio of the cylindrical bluff bodies with different lengths.

Cylinder Length	65 mm	94 mm	178 mm
AR	3.4	5.0	9.4
$b/L_{c}$	0.32	0.22	0.12
$m^{*}$	∼570	∼430	∼210

**Table 3:** Natural frequencies and mass ratios of the harvesters equipped with cylindrical bluff bodies of different masses.

Cylinder Mass	5 gr	7.5 gr	10 gr	12.5 gr	15 gr
$f_{n}$ (Hz)	20.7	18.1	16.8	15.1	14.1
$m^{*}$	∼130	∼170	∼210	∼250	∼290

## Data Availability

Dataset available on request from the authors.

## Nomenclature

$x_{1}$	patch start from clamp
$x_{2}$	patch end from clamp
*L*	cantilever length
*b*	cantilever width
*D*	cylinder external diameter
$L_{c}$	cylinder length
$f_{s}$	shedding frequency
*U*	wind speed
*t*	time variable
*M*	bending moment of the cantilever
$c_{s}$	equivalent coefficient of strain rate damping
*I*	equivalent moment of inertia of the composite cross section
$c_{a}$	viscous air damping coefficient
*m*	mass per unit length of the beam
*F*	generic force applied to the cantilever tip
$F_{a}$	harmonic lift applied to the cylinder axis
δ	Dirac delta function
EI	bending stiffness of the composite cross section of the cantilever
*H*	Heaviside function
$e_{31}$	piezoelectric constant
$h_{p}$	thickness of the piezoelectric layer
$h_{b}$	position of the bottom of the piezoelectric layer from the neutral axis
$h_{c}$	position of the top of the piezoelectric layer from the neutral axis
$h_{pc}$	distance of the center of the piezoelectric patch cross section from the neutral axis
$\Psi_{n}$	*n*th mode of vibration
$\eta_{n}$	*n*th modal displacement
$m_{n}$	modal mass of the *n*th mode of vibration
$c_{n}$	modal damping coefficient of the *n*th mode of vibration
$k_{n}$	modal stiffness of the *n*th mode of vibration
$\varphi_{n}$	forward modal coupling coefficient *n*th mode of vibration
$f_{n}$	modal force of the *n*th mode of vibration
$\zeta_{n}$	modal damping ratio of the *n*th mode of vibration
Γ	surface of the positive electrode

D	electric displacement
n	unit normal vector of Γ
$S_{1}$	strain within the piezoelectric layer
$E_{3}$	transverse component of the electric field
$D_{3}$	transverse component of electric displacement
$e_{31}$	piezoelectric stress charge coefficient
$\varepsilon_{33}^{S}$	permittivity at constant strain
*v*	electric voltage
$v_{0}$	open-circuit voltage
*i*	output current
$C_{p}$	piezoelectric patch capacitance
$E_{e}$	electric potential energy stored in the harvester
$E_{e_{avg}}$	average electric potential energy stored in the harvester
$v_{0_{RMS}}$	root mean square (RMS) value of the open-circuit voltage
ρ	fluid density
$C_{L}$	lift coefficient
$\tau_{1}$	torque applied to the tip
$\chi_{n}$	modal constant of the *n*th mode
$\omega_{1}$	natural angular frequency of the first mode of the coupled system
$\omega_{s}$	angular frequency of vortex shedding
SG	Skop–Griffin number
St	Strouhal number
$m^{*}$	mass ratio between the vibrating mass and the mass of fluid having the same volume of the cylinder
